# Association of dietary inflammatory index with sarcopenia in asthmatic patients: a cross-sectional study

**DOI:** 10.3389/fnut.2023.1215688

**Published:** 2023-08-31

**Authors:** Shuqiong Lin, Xia Su, Liqun Chen, Zhiming Cai

**Affiliations:** ^1^Zhangzhou Affiliated Hospital of Fujian Medical University, Zhangzhou, China; ^2^Longyan First Affiliated Hospital of Fujian Medical University, Longyan, China

**Keywords:** asthma, sarcopenia, dietary inflammatory index, diet, NHANES

## Abstract

**Background:**

Sarcopenia is a complication of asthma, and asthmatics with sarcopenia are at an increased risk of poor prognosis. Anti-inflammatory intervention promising as an effective measure to prevent sarcopenia among patients with asthma. Diet is an important way to regulate inflammation throughout the body. The dietary inflammatory index (DII) is an index that assesses an individual’s overall dietary inflammatory potential. The relationship between DII and sarcopenia among patients with asthma is not clear.

**Objective:**

To examine the correlation between DII and the sarcopenia among individuals with asthma.

**Methods:**

The National Health and Nutrition Examination Survey (NHANES) was the data source utilized in this study, spanning two time periods from 1999 to 2006 and 2011 to 2018. The study encompassed 3,389 participants in total. DII was calculated using the results of the participants’ 24-h dietary recall interviews. Patients were categorized into three groups based on the DII tertile: T1 group (*n* = 1,130), T2 group (*n* = 1,129), and T3 group (*n* = 1,130). Logistic regression analysis, taking into account the NHANES recommended weights, was performed to assess the relationship between DII and sarcopenia.

**Results:**

After full adjustment, there was a significant positive correlation between DII levels and the risk of sarcopenia in asthmatic patients (OR: 1.27, 95% CI: 1.13–1.42, *p* < 0.001). Compared with T1 group, T3 group had higher risk of sarcopenia (T2: OR: 1.39, 95%CI: 0.88–2.18, *p* = 0.157; T3: OR: 2.37, 95%CI: 1.47–3.83, *p* < 0.001).

**Conclusion:**

There was a significant positive correlation between DII and the risk of sarcopenia.

## Introduction

Bronchial asthma, a chronic disease affecting 330 million people worldwide, is characterized by airway inflammation and hyperresponsiveness (1, 2), with asthma patients having abnormally elevated levels of multiple inflammatory factors such as interleukin-1 (IL-1), IL-6, IL-17, and tumor necrosis factor-α (TNF-α) (3). These inflammatory factors can cause skeletal muscle loss through a variety of pathways [such as nuclear factor κb (NF-κB)], leading to sarcopenia (4). Sarcopenia is an emerging global health problem characterized by the gradual decline of skeletal muscle mass and can lead to adverse complications (5). About 3% of the world’s population suffers from sarcopenia (6), and individuals with asthma have a higher prevalence of this condition. According to research, the prevalence of sarcopenia in individuals with asthma can be as high as 17.6%, and approximately 5.5% of asthmatic are affected by severe sarcopenia (7). Furthermore, individuals with both asthma and sarcopenia were also at higher risk of osteoporosis, decreased lung function, and depression (7), and asthma patients with sarcopenia experience more severe shortness of breath and airway obstruction (8). Therefore, it is necessary to prevent sarcopenia among patients with asthma.

Inflammation is one of the causes of sarcopenia in asthmatic patients, which make anti-inflammatory intervention promising as an effective measure to prevent sarcopenia among individuals with asthma. Regulating inflammation can be achieved through dietary. Many components of the diet have been linked to inflammation. For instance, consuming higher amounts of cholesterol and carbohydrates has been found to contribute to increased levels of inflammation markers in the body (9–11). Increasing intake of dietary fiber, folic acid, and garlic has a positive effect on reducing levels of markers of inflammation (12–14). Nonetheless, due to the diversity of daily diets and the complex nature of individual food components, it remains a challenge to gage a person’s comprehensive dietary inflammation level. Therefore, previous studies developed a dietary inflammation index (DII) that takes into account the anti-inflammatory and pro-inflammatory properties of different food components. This enables a more comprehensive evaluation of the overall extent of dietary inflammation in individuals (15). Previous study has shown that asthmatic patients with elevated DII levels had a higher risk of experiencing all-cause mortality (16). However, to our knowledge, few studies have investigated whether there is an association between DII and sarcopenia in asthmatic patients.

The objective of this investigation was to examine the correlation between DII and the sarcopenia among individuals with asthma, and to provide some valuable insights for the prevention of sarcopenia in patients with asthma.

## Methods

### Study population

This study utilized data from the National Health and Nutrition Examination Survey (NHANES), which is sponsored by the National Center for Health Statistics (NCHS). NHANES is a sample survey conducted annually across the United States that surveys 5,000 Americans about their physical health. Two years is a cycle, and about 10,000 people are surveyed in each cycle. Through a sampling weighted analysis, the study cohort represents the entire population of the United States.

**Figure 1:**
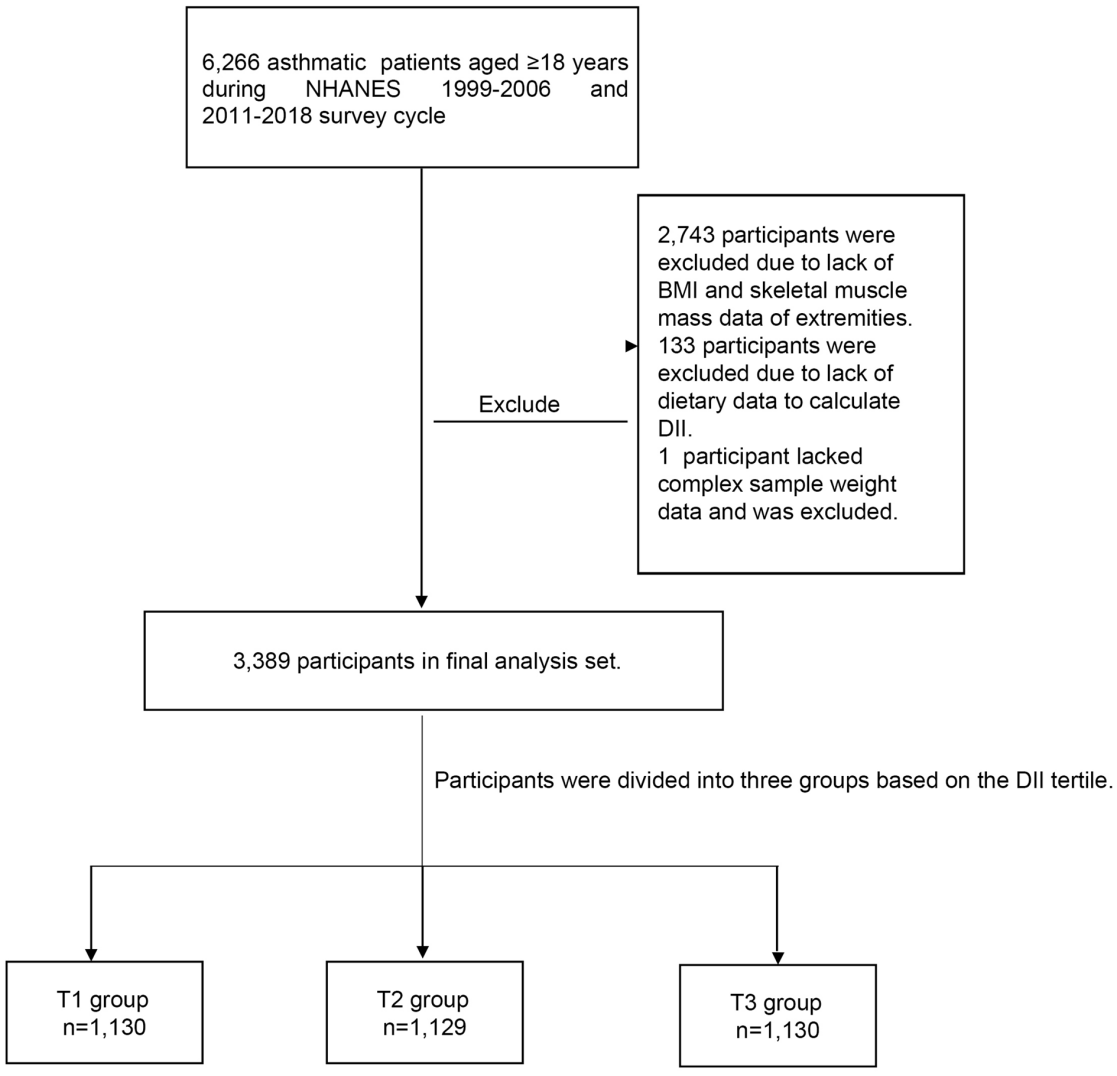
Flowchart of the study design.

Due to the lack of records on skeletal muscle mass measurements during 2007–2010, the scope of the study was confined only to those who took part in the survey between 1999–2006 and 2011–2018. Among the NHANES participants from 1999–2006 to 2011–2018, a total of 80,630 people participated in the survey over the 8 cycles, and there were 6,266 asthma patients aged ≥18. In addition, we excluded 2,743 individuals who did not possess sufficient skeletal muscle mass or body mass index (BMI) information, 133 individuals who did not have dietary data available for DII calculation, and one participant who was missing dietary weight data. Finally, a total of 3,389 individuals were included in the study (Figure 1).

### Definition of asthma

According to the NHANES questionnaire, “Have you been told by a doctor that you have asthma?” patients who answered yes were considered asthmatic.

### Primary outcome

The primary outcome is sarcopenia. According to the recommendations of Foundation for National Institutes of Health Osteoarthritis Biomarkers study (FNIH), men with a sarcopenia index of less than 0.789 and women with a sarcopenia index of less than 0.512 are considered to have sarcopenia, the sarcopenia index = total appendicular skeletal muscle mass (in kg)/BMI (kg/m^2^) (17). NHANES official staff measured limb lean body mass with dual-energy X-ray absorptiometry (DXA), and calculated appendical skeletal muscle mass with the sum of limb lean body mass. In the NHANES files “dxdllle,” “dxdllle,” “dxdrale,” and “dxdrlle,” the specific values of limb lean body mass are recorded.^1^

### Calculation of the DII

The collection of dietary information in NHANES was obtained by conducting 24-h dietary recall interviews at mobile inspection centers. In our study, we used 28 food parameters to calculate the DII. The foods included carbohydrates, proteins, total fats, alcohol, fibers, cholesterol, saturated fatty acids (SFAs), monounsaturated fatty acids (MUFAs), polyunsaturated fatty acids (PUFAs), omega-3 fatty acids, omega-6 fatty acids, vitamin A, vitamin B1, vitamin B2, vitamin B6, vitamin B12, vitamin C, vitamin D, vitamin E, folic acid, niacin, magnesium, zinc, iron, selenium, beta-carotene, caffeine, and energy (15). Since only these 28 food items in NHANES can be used to calculate DII, the total DII for only 28 foods was calculated in this study. But it has been established in previous studies that the predictive efficacy of DII remains unaffected even when calculated using only these 28 food items (18). The cut points and the scoring system were shown in Supplementary Table 1.

### Confounding variable

Participants in the study self-reported their age, gender, race, smoking status, drinking status, height, and weight. BMI was calculated using their height and weight measurements. Triglycerides (TG) and total cholesterol (TC) were measured using a Hitachi Model 704 multichannel analyzer by the Coulston Foundation (Alamogordo, NM, United States) and a collaborating laboratory (Ottumwa, IA, United States). C-reactive protein (CRP) levels were quantified by the University of Washington (Seattle, WA, United States) using the latex-enhanced turbidity method after NHANES staff collected blood samples. Detailed procedures can be found in the NHANES official website.^2^

Forced expiratory volume in the first second (FEV1)/forced vital capacity (FVC) measurements were obtained using a spirometer, which is a device that measures lung function. NHANES Respiratory Health Spirometer Procedure Manual^3^ provides detailed procedures for obtaining spirometer measurements.

The patient’s current medication use was determined based on their self-reported prescriptions. Anti-asthmatic agents, such as mast cell stabilizers, selective phosphodiesterase-4 inhibitors, leukotriene regulators, and inhaled corticosteroids, were considered. The identification of hypertension, diabetes mellitus (DM), chronic kidney disease (CKD), and cardiovascular disease (CVD) relied on positive answers to the inquiry “Has a medical practitioner or any other healthcare professional ever informed you about your condition of hypertension, DM, CKD, and CVD?” Patients with FEV1/FVC < 0.7 after inhaling beta 1 adrenergic bronchodilator medication were considered to have chronic obstructive pulmonary disease (COPD).

### Method of grouping

Based on the DII tertile, the patients were categorized into three groups: T1 group (DII < 0.9), T2 group (0.9 ≤ DII < 2.7), and T3 group (DII ≥ 2.7).

### Statistical analyses

According to the weighted recommended by the NHANES, participants were assigned corresponding sampling weights, and all statistical analyses used were calculated by sampling weights. The mean (weighted) and standard deviation (weighted) were used to represent continuous variables, while counts and percentages (weighted) were used to represent categorical variables. We employed ANOVA to assess differences in continuous variables across various groups, while χ^2^ test was leveraged to evaluate disparities in categorical variables.

In order to assess the correlation between the DII and sarcopenia among patients with asthma, logistic regression analysis was employed. NHANES weights were utilized to obtain estimates and probabilities. Model 1 represented the unadjusted analysis, while Model 2 accounted for age, gender, and race as adjustments. Model 3 was comprehensively adjusted to include other potential confounders such as age, gender, race/ethnicity, smoking habits, drinking habits, BMI, TG, TC, hypertension, DM, CKD, COPD, and anti-asthmatic agents. In order to investigate possible non-linear connections between DII and the sarcopenia among patients with asthma, a regression cubic spline (RCS) was utilized. Furthermore, a stratified analysis was conducted based on age, gender, BMI, smoking, COPD, hypertension, DM, and anti-asthmatic agents.

Data analyses were carried out using the Survey package in R Studio (version 4.2.2). Statistical significance was determined based on a *p* value of less than 0.05.

## Results

### Participant characteristics

The study encompassed 3,389 participants in total. Participants were 39.0 (0.4) years old on average and were more likely to be female [*n* = 1,870(55.1%)]. Based on their DII tertile, the participants were categorized into three distinct groups: T1 (*n* = 1,130), T2 (*n* = 1,129), and T3 (*n* = 1,130). There were statistical differences in the proportion of female [T1: 484 (41.5%) vs. T2: 631 (56.7%) vs. T3: 755 (70.4%); *p* < 0.001], drinkers [T1: 823 (83.5%) vs. T2: 714 (76.3%) vs. T3: 672 (72.0%); *p* < 0.001], DM [T1: 101 (5.8%) vs. T2: 134 (9.3%) vs. T3: 139 (11.1%); *p* = 0.001], and COPD [T1: 70 (7.4%) vs. T2: 88 (7.7%) vs. T3: 117 (11.7%); *p* = 0.042] among the three groups. And there were statistical differences in BMI (T1: 27.7 ± 6.4 kg/m^2^ vs. T2: 28.6 ± 6.8 kg/m^2^ vs. T3: 29.4 ± 7.4 kg/m^2^; *p* < 0.001) and CRP (T1: 0.3 ± 0.6 mg/dL vs. T2: 0.4 ± 0.6 mg/dL vs. T3: 0.6 ± 0.8 mg/dL; *p* < 0.001) among the three groups. Among the three groups, there were no statistical difference in terms of age, smoking status, TG and TC levels, use of anti-asthmatic medication, presence of hypertension, and CKD (Table 1).

**Table 1:** Baseline study population characteristics (weighted).

Variable	Total (*n* = 3,389)	T1 group (*n* = 1,130)	T2 group (*n* = 1,129)	T3 group (*n* = 1,130)	*p* value
DII	1.4 ± 1.9	−0.6 ± 1.2	1.8 ± 0.5	3.4 ± 0.5	< 0.001^+^
Age, years	39.0 ± 14.6	38.9 ± 14.3	39.8 ± 15.1	38.3 ± 14.6	0.248^+^
Female, *n* (%)	1,870 (55.1)	484 (41.5)	631 (56.7)	755 (70.4)	< 0.001^#^
Race, *n* (%)					0.001^#^
Mexican American	413 (6.1)	134 (6.0)	147 (6.4)	132 (5.9)	
Non-Hispanic black	834 (12.6)	234 (9.4)	296 (15.3)	304 (13.7)	
Non-Hispanic white	1,583 (69.2)	569 (73.9)	491 (64.3)	523 (68.3)	
Other Hispanic	239 (5.8)	65 (4.3)	93 (7.2)	81 (6.3)	
Other race	320 (6.3)	128 (6.4)	102 (6.8)	90 (5.8)	
Smoke status, *n* (%)	1,452 (48.8)	461 (46.2)	477 (47.9)	514 (53.1)	0.087^#^
Drink status, *n* (%)^*^	2,209 (77.8)	823 (83.5)	714 (76.3)	672 (72.0)	< 0.001^#^
BMI, kg/m^2^	28.5 ± 6.9	27.7 ± 6.4	28.6 ± 6.8	29.4 ± 7.4	< 0.001^+^
TG, mmol/L	1.7 ± 2.3	1.7 ± 3.0	1.7 ± 1.8	1.6 ± 1.5	0.593^+^
TC, mmol/L	5.0 ± 1.3	5.0 ± 1.2	5.1 ± 1.2	5.0 ± 1.2	0.599^+^
CRP, mg/dL	0.4 ± 0.7	0.3 ± 0.6	0.4 ± 0.6	0.6 ± 0.8	< 0.001^+^
Anti-asthmatic agents, *n* (%)	394 (12.3)	127 (12.0)	124 (10.6)	143 (14.5)	0.159^#^
DM, *n* (%)	374 (8.5)	101 (5.8)	134 (9.3)	139 (11.1)	0.001^#^
Hypertension, *n* (%)	1,105 (29.9)	346 (27.4)	387 (31.3)	372 (31.6)	0.187^#^
CKD, *n* (%)	397 (10.0)	109 (8.6)	149 (10.3)	139 (11.2)	0.293^#^
COPD, *n* (%)	275 (8.8)	70 (7.4)	88 (7.7)	117 (11.7)	0.042^#^
Sarcopenia, *n* (%)	347 (10.2)	85 (5.8)	112 (7.6)	150 (12.2)	< 0.001^#^

Values are, *n* (%) or mean ± SD. ^+^ANOVA test; ^#^*χ*^2^ test. DII, dietary inflammation index; BMI, body mass index; CRP: C-reactive protein; TG, triglyceride; TC, total cholesterol; DM, diabetes; CKD, chronic kidney disease; COPD, chronic obstructive pulmonary disease.

* The average number of drinks consumed per day over the past 12 months.

### Relationship between DII and sarcopenia

**Figure 2:**
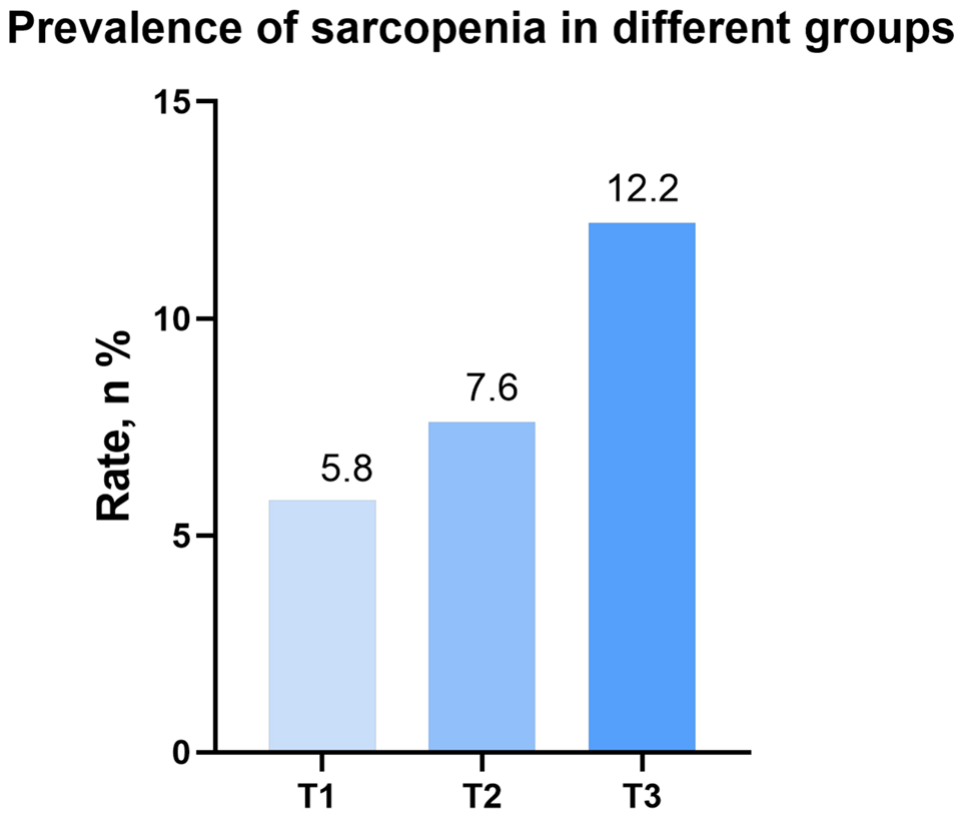
Prevalence of sarcopenia in different groups (weighted). DII, dietary inflammation index; BMI, body mass index.

As shown in Figure 2, individuals with higher levels of DII exhibited a greater prevalence of sarcopenia (T1: 5.8% vs. T2: 7.6% vs. T3: 12.2%). Through the implementation of univariate logistic regression analysis, it was found that a significant positive correlation between DII and the risk of sarcopenia [odds ratio (OR): 1.26, 95% CI: 1.15–1.39, *p* < 0.001; Model 1]. In contrast to the T1 group, only the T3 group had a higher risk of sarcopenia (OR: 2.26, 95%CI: 1.56–3.29; *p* < 0.001; Model 1). After adjusting for age, gender and race, the positive correlation between DII and sarcopenia remained statistically significant (OR: 1.35, 95% CI: 1.21–1.49, *p* < 0.001; Model 2). Participants in T3 group had a higher risk of sarcopenia (OR: 2.87, 95%CI: 1.93–4.26; *p* < 0.001; Model 2). Moreover, when accounting for all potential confounders, the relationship between DII and sarcopenia remained statistically significant (OR: 1.27, 95% CI: 1.13–1.42, *p* < 0.001; Model 3). Furthermore, participants in the T3 group continued to exhibit a higher risk of sarcopenia (OR: 2.37, 95%CI: 1.47–3.83; *p* < 0.001; Model 3). Additional details can be found in Table 2.

**Table 2:** The association between DII and sarcopenia (weighted).

Variable	Model 1		Model 2		Model 3	
	OR (95%CI)	*p* value	OR (95%CI)	*p* value	OR (95%CI)	*p* value
Continuous variables						
DII	1.26 (1.15–1.39)	<0.001	1.35 (1.21,1.49)	<0.001	1.27 (1.13,1.42)	<0.001
Categorical variable						
T1 group	Ref		Ref		Ref	
T2 group	1.35 (0.93,1.95)	0.110	1.41 (0.97,2.04)	0.068	1.39 (0.88,2.18)	0.157
T3 group	2.26 (1.56,3.29)	<0.001	2.87 (1.93,4.26)	<0.001	2.37 (1.47,3.83)	<0.001

Model 1: Not adjusted. Model 2: Adjusted by age, gender, and race. Model 3: Adjusted by age, gender, race, smoke, drink, BMI, TG, TC, hypertension, DM, CKD, COPD, and anti-asthmatic agents.

### Regression cubic splines

**Figure 3:**
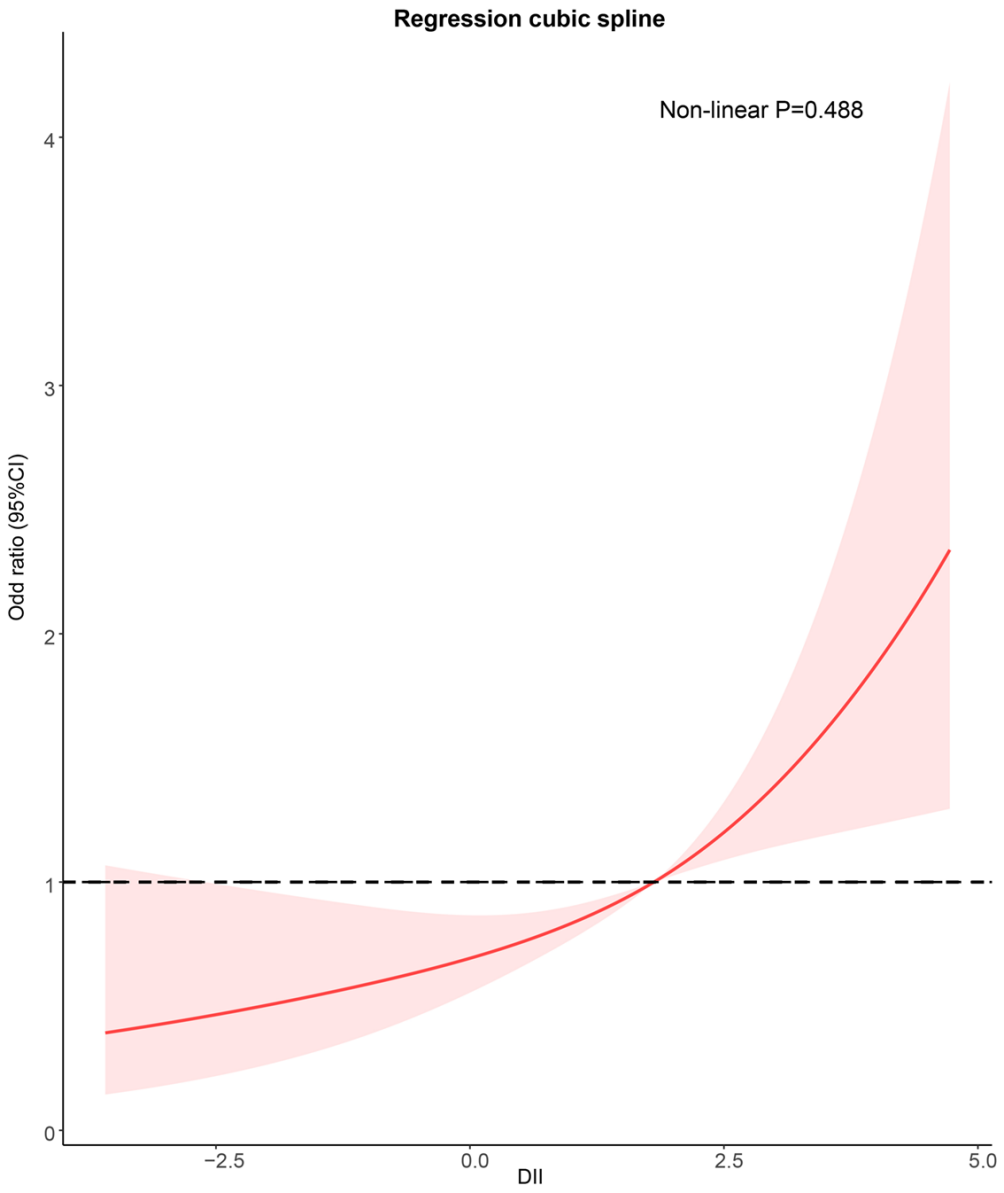
Potential nonlinear relationship between DII and sarcopenia (weighted). Adjusted for age, sex, race/ethnicity, smoking, drinking, BMI, TG, TC, hypertension, DM, CKD, COPD, and anti-asthma medications. DII, dietary inflammation index; BMI, body mass index; TG, triglyceride; TC, total cholesterol; DM, diabetes; CKD, Chronic kidney disease; and COPD: Chronic obstructive pulmonary disease.

Regression cubic spline adjusted for Model 3 showed that DII was positively correlated with the risk of sarcopenia in asthmatic patients. No nonlinear relationship was observed between DII and sarcopenia (Non-linear *p* = 0.488; Figure 3).

### Subgroup analysis

**Figure 4:**
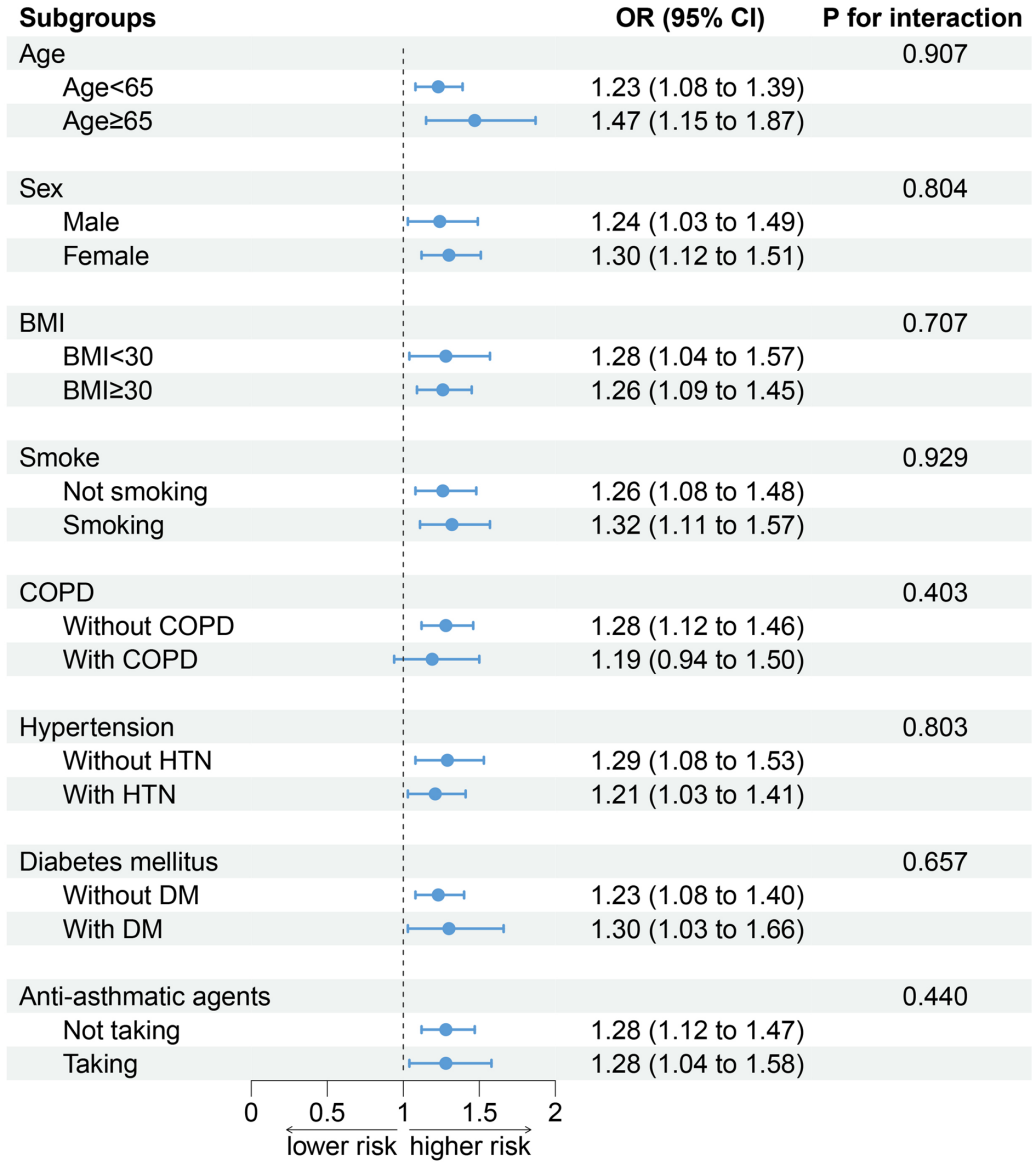
Association between DII and sarcopenia by selected subgroups (weighted). Adjusted for age, sex, race/ethnicity, smoking, drinking, BMI, TG, TC, hypertension, DM, CKD, COPD, and anti-asthma medications. When stratified, stratified variables are not adjusted. DII, dietary inflammation index; BMI, body mass index; TG, triglyceride; TC, total cholesterol; DM, diabetes; CKD, chronic kidney disease; and COPD: chronic obstructive pulmonary disease.

No alteration was observed in the association between DII and sarcopenia when conducting a stratified analysis according to age (*p* for interaction = 0.907), sex (*p* for interaction = 0.804), BMI (*p* for interaction = 0.707), smoking (*p* for interaction = 0.929), COPD (*p* for interaction = 0.403), hypertension (*p* for interaction = 0.803), DM (*p* for interaction = 0.657), and antiasthmatic drugs (*p* for interaction = 0.440; Figure 4).

## Discussion

A total of 3,389 asthmatic patients were enrolled in this study. Our findings indicate a noteworthy positive correlation between the DII and sarcopenia among asthmatic patients. After stratification by age, sex, BMI, smoking, COPD, hypertension, DM and antiasthmatic drugs, no substantial alteration in the correlation between DII and sarcopenia was observed.

According to a meta-analysis, an elevated DII level was found to be significantly linked to the occurrence and progression of chronic illnesses, including CVD, cancer, and respiratory disease (19). In a study conducted on individuals with asthma, Han et al. (20) demonstrated that a higher DII level was connected to an elevated risk of wheezing. Additionally, Yuan et al. (16) observed that asthmatic patients with elevated DII levels had a higher risk of experiencing all-cause mortality. Nevertheless, the relationship between DII and sarcopenia in asthmatic individuals remains poorly investigated.

Several studies have demonstrated that increased levels of DII are a risk factor for the onset of sarcopenia in older adults (21–23). Similarly, heightened DII have also been linked to an elevated risk of sarcopenia in patients with DM, hypertension, Crohn’s disease, and CKD (24–26). It is well-established that aging is associated with a chronic state of low-grade inflammation in older adults (27). Many studies observed elevated levels of proinflammatory factors in the blood of people with hypertension and diabetes (28, 29). Additionally, both Crohn’s disease and CKD are characterized by persistent inflammation (30, 31). Like these population, asthma is also a chronic inflammatory disease (32), and studies have shown that people with asthma were more susceptible to the pro-inflammatory effects of dietary factors when compared to those without asthma (20). Furthermore, it should be noted that numerous asthma drugs have been found to contribute to the development of sarcopenia (33, 34). Sarcopenia in patients may exacerbate their asthma symptoms, thereby necessitating more anti-asthma medication, leading to a vicious cycle. Therefore, as with the elderly, DM, hypertension, Crohn’s disease, and CKD patients, the prevention of sarcopenia in asthma patients is also worthy of attention. Our study fills a gap in this area by using DII to assess the risk of sarcopenia among patients with asthma. Our findings suggest that DII was significantly correlated with the risk of sarcopenia in patients with asthma. Following the adjustment for potential confounders, the relationship between the two did not change significantly. In addition, stratified results by age, sex, BMI, smoking, hypertension, diabetes, and anti-asthma medications did not change significantly, and no interaction was observed between DII and these variables.

The mechanism by which a pro-inflammatory diet induces sarcopenia in asthmatic patients is unclear, but previous studies have provided some theoretical evidence. The study suggests that trans fatty acids have been shown to increase airway inflammation and circulating CRP levels in asthma patients (35, 36). In addition, inflammatory factors such as platelet-derived growth factor (PDGF), interferon-γ (IFN-γ), IL-1, and TNF-α were significantly increased in mice with high cholesterol intake (37). Excessive intake of refined carbohydrates increases circulating TNF-α levels via the NF-κB pathway (38). In contrast, ingestion of PUFAs produces anti-inflammatory mediators and inhibits the production of pro-inflammatory factors (39). A randomized controlled trial confirmed that increasing MUFAs intake helped lower CRP and IL-6 (40). TNF-α can induce sarcopenia through pyrodeath mediated by Gasdermins (41). IL-6 can be indicated by glycoprotein 130 (gp130)/Janus kinase (JAK)/Signal Transducer and Activator of Transcription (STAT)/Suppressor of Cytokine Signaling (SOCS) pathways increase protein degradation and decrease protein synthesis to mediate skeletal muscle atrophy (42). Britta Walling-Larsson et al. (43) indicated that CRP can lead to downregulation of serine/threonine kinase Akt promoting muscle atrophy. PDGF can cause muscle fibrosis and hinder muscle repair by activating RHO-associated kinases (44). IFN-γ interferes with the differentiation of muscle satellite cells, leading to the accumulation of muscle satellite cells and the loss of skeletal muscle mass (4). Therefore, the loss of muscle mass caused by a pro-inflammatory diet may result from a number of pathways.

It should be emphasized that the correlation between protein and inflammation is not solely linked to consumption but also to the origin of protein. Animal-derived proteins lead to increased levels of pro-inflammatory factors, while plant-derived proteins help reduce inflammation throughout the body (36). A recent study showed that intake of animal protein increases the risk of developing frailty (a phenotypic manifestation of sarcopenia) (45–47), while increasing plant protein intake was linked with a lower possibility of developing frailty (48, 49). Therefore, when consuming protein, choosing to consume more plant protein may help prevent sarcopenia.

As mentioned above, the major pro-inflammatory parameters in DII, such as carbohydrates, cholesterol, trans fatty acids and animal protein, were strongly associated with the onset and development of sarcopenia. Thus, reducing the consumption of these pro-inflammatory components may yield beneficial effects in preventing sarcopenia. Increasing the intake of anti-inflammatory food components such as PUFAs, MUFAs, and plant proteins in DII can reduce the level of inflammation throughout the body and is one of the potential interventions to prevent sarcopenia. Therefore, we recommend that people with asthma reduce their intake of pro-inflammatory food components in the DII dietary pattern and increase their intake of anti-inflammatory food components. Although a causal relationship between DII and sarcopenia in asthmatic patients need to be further confirmed in randomized clinical trials. However, prior research has established that patients with asthma experienced a decrease in wheezing attacks and all-cause mortality by maintaining low levels of DII. Therefore, despite being a cross-sectional study, we still recommend that patients with asthma adopt a healthy low-DII dietary pattern.

### Limitations

There are certain constraints inherent in our study. Initially, due to its cross-sectional design, we can only establish a correlation between the DII and sarcopenia in asthmatic patients, rather than establish a causal relationship. Secondly, relying solely on self-reported questionnaires to diagnose asthmatics may underestimate the number of asthmatics. Third, assessing DII solely on the basis of 24-h dietary recall may not be representative of patients’ long-term dietary patterns, and further clinical randomized controlled trials are needed to further corroborate our results.

## Conclusion

There was a significant positive correlation between DII and the risk of sarcopenia. However, additional randomized controlled trials are necessary to establish a definitive causal connection between the DII and the onset of sarcopenia.

## Data availability statement

The raw data supporting the conclusions of this article will be made available by the authors, without undue reservation.

## Ethics statement

The studies involving humans were approved by National Center for Health Statistics. The studies were conducted in accordance with the local legislation and institutional requirements. The human samples used in this study were acquired from this study utilized data from the National Health and Nutrition Examination Survey (NHANES), which is sponsored by the National Center for Health Statistics (NCHS). Written informed consent for participation was not required from the participants or the participants’ legal guardians/next of kin in accordance with the national legislation and institutional requirements.

## Author contributions

ZC designed the research and is the guarantor of this work and, as such, had full access to all the data in the study and takes responsibility for the integrity of the data and the accuracy of the data analysis. SL conducted the analysis and wrote the first draft of the paper. SL, XS, LC, and ZC revised the manuscript. All authors contributed to the article and approved the submitted version.

## Conflict of interest

The authors declare that the research was conducted in the absence of any commercial or financial relationships that could be construed as a potential conflict of interest.

## Publisher’s note

All claims expressed in this article are solely those of the authors and do not necessarily represent those of their affiliated organizations, or those of the publisher, the editors and the reviewers. Any product that may be evaluated in this article, or claim that may be made by its manufacturer, is not guaranteed or endorsed by the publisher.

## Figures and Tables

**Table:** 

IL	Interleukin
TNF-α	Tumor necrosis factor-α
NF-κB	Nuclear factor κb
DII	Dietary inflammation index
NHANES	National Health and Nutrition Examination Survey
NCHS	National Center for Health Statistics
FNIH	Foundation for National Institutes of Health Osteoarthritis Biomarkers study
DXA	Dual-energy X-ray absorptiometry
BMI	Body mass index
SFAs	Saturated fatty acids
MUFAs	Monounsaturated fatty acids
PUFAs	Polyunsaturated fatty acids
TG	Triglycerides
TC	Total cholesterol
DM	Diabetes mellitus
CKD	Chronic kidney disease
CVD	Cardiovascular disease
FEV1	Forced expiratory volume in the first second
FVC	Forced vital capacity
COPD	Chronic obstructive pulmonary disease
ANOVA	Analysis of variance
RCS	Regression cubic spline
CRP	C-reactive protein
PDGF	Platelet-derived growth factor
IFN-γ	Interferon-γ
gp130	Glycoprotein 130
JAK	Janus kinase
STAT	Signal transducer and activator of transcription
SOCS	Suppressor of cytokine signaling

## Footnotes

^1^ https://wwwn.cdc.gov/nchs/data/nhanes/dxa/dxx_b.pdf

^2^ https://wwwn.cdc.gov/nchs/data/nhanes/2009-2010/manuals/lab.pdf

^3^ https://wwwn.cdc.gov/nchs/data/nhanes/2007-2008/manuals/spirometry.pdf
