# Supplementary material for: Association of dietary inflammatory index with sarcopenia in asthmatic patients: a cross-sectional study
Source: Front Nutr. 2023 Aug 31;10:1215688. doi: 10.3389/fnut.2023.1215688 (PMC10501140; doi:10.3389/fnut.2023.1215688)
Supplement: Supplementary file 1 [file Table_1.DOCX]

**Supplementary table 1.** **Anti-inflammatory and proinflammatory parameters of various foods**

| **Food parameter** | **Weighted number of articles** | **Raw inflammatory effect score** | **Overall inflammatory effect score** | **Global daily mean intake‡ (units/d)** | **SD** |
| --- | --- | --- | --- | --- | --- |
| Carbohydrate(g) | 211 | 0·109 | 0·097 | 272·2 | 40·0 |
| Proteins(g) | 102 | 0·049 | 0·021 | 79·4 | 13·9 |
| Total fat (g) | 443 | 0·298 | 0·298 | 71·4 | 19·4 |
| Alcohol (g) | 417 | –0·278 | –0·278 | 13·98 | 3·72 |
| Cholesterol (mg) | 75 | 0·347 | 0·110 | 279·4 | 51·2 |
| Fibre (g) | 261 | –0·663 | –0·663 | 18·8 | 4·9 |
| Saturated fat (g) | 205 | 0·429 | 0·373 | 28·6 | 8·0 |
| MUFA (g) | 106 | –0·019 | –0·009 | 27·0 | 6·1 |
| PUFA (g) | 4002 | –0·337 | –0·337 | 13·88 | 3·76 |
| omega-3 fatty acids(g) | 2588 | –0·436 | –0·436 | 1·06 | 1·06 |
| omega-6 fatty acids(g) | 924 | –0·159 | –0·159 | 10·80 | 7·50 |
| Vitamin A (RE) | 663 | –0·401 | –0·401 | 983·9 | 518·6 |
| Vitamin B1 (mg) | 65 | –0·354 | –0·098 | 1·70 | 0·66 |
| Vitamin B2 (mg) | 22 | –0·727 | –0·068 | 1·70 | 0·79 |
| Vitamin B6 (mg) | 227 | –0·379 | –0·365 | 1·47 | 0·74 |
| Vitamin B12 (mg) | 122 | 0·205 | 0·106 | 5·15 | 2·70 |
| Vitamin C (mg) | 733 | –0·424 | –0·424 | 118·2 | 43·46 |
| Vitamin D (μg) | 996 | –0·446 | –0·446 | 6·26 | 2·21 |
| Vitamin E (mg) | 1495 | –0·419 | –0·419 | 8·73 | 1·49 |
| Folic acid (μg) | 217 | –0·207 | –0·190 | 273·0 | 70·7 |
| Niacin (mg) | 58 | –1·000 | –0·246 | 25·90 | 11·77 |
| Magnesium (mg) | 351 | –0·484 | –0·484 | 310·1 | 139·4 |
| Zinc (mg) | 1036 | –0·313 | –0·313 | 9·84 | 2·19 |
| Iron (mg) | 619 | 0–032 | 0·032 | 13·35 | 3·71 |
| Selenium (μg) | 372 | –0·191 | –0·191 | 67·0 | 25·1 |
| β-Carotene (μg) | 401 | –0·584 | –0·584 | 3718 | 1720 |
| Caffeine (g) | 209 | –0·124 | –0·110 | 8·05 | 6·67 |
| Energy (kcal) | 245 | 0·180 | 0·180 | 2056 | 338 |
